# Crosstalk between Tumor Cells and Macrophages in Stroma Renders Tumor Cells as the Primary Source of MCP-1/CCL2 in Lewis Lung Carcinoma

**DOI:** 10.3389/fimmu.2015.00332

**Published:** 2015-06-26

**Authors:** Teizo Yoshimura, Mingyong Liu, Xin Chen, Liangzhu Li, Ji Ming Wang

**Affiliations:** ^1^Laboratory of Molecular Immunoregulation, Cancer and Inflammation Program, Center for Cancer Research, National Cancer Institute, Frederick, MD, USA; ^2^Department of Spine Surgery, Daping Hospital, Third Military Medical University, Chongqing, China; ^3^State Key Laboratory of Quality Research in Chinese Medicine, Institute of Chinese Medical Sciences, University of Macau, Macao, China; ^4^Basic Science Program, Frederick National Laboratory for Cancer Research, Leidos Biomedical Research, Inc., Frederick, MD, USA; ^5^Engineering Research Center for Cell and Therapeutic Antibody of Ministry of Education, School of Pharmacy, Shanghai Jiaotong University, Shanghai, China

**Keywords:** monocytes/macrophages, chemokines, inflammation, tumor microenvironment, lung cancer

## Abstract

The chemokine MCP-1/CCL2 is produced by a variety of tumors and plays an important role in cancer progression. We and others previously demonstrated that the primary source of MCP-1 in several mouse tumors, including 4T1 breast cancer, M5076 sarcoma, and B16 melanoma, was stromal cells. In the present study, we identified that tumor cells were the primary source of MCP-1 in Lewis lung carcinoma (LLC), because MCP-1 mRNA was highly expressed in tumors grown in both wild type (WT) and MCP-1^−/−^ mice with elevated serum MCP-1 levels. Since LLC cells isolated from tumors expressed low levels of MCP-1 *in vitro*, it appeared that the tumor–stromal cell interaction in a tumor microenvironment increased MCP-1 expression in LLC cells. In fact, co-culture of LLC cells with normal mouse peritoneal macrophages or normal lung cells containing macrophages increased MCP-1 expression by LLC cells. Macrophages from TNFα^−/−^ mice failed to activate LLC cells and anti-TNFα neutralizing antibody abolished the effect of WT macrophages on LLC cells. When LLC cells were transplanted into TNFα^−/−^ mice, the levels of MCP-1 mRNA in tumors and serum MCP-1 levels were markedly lower as compared to WT mice, and importantly, tumors grew more slowly. Taken together, our results indicate that TNFα released by tumor cell-activated macrophages is critical for increased MCP-1 production by tumors cells. Thus, disruption of tumor–stromal cell interaction may inhibit tumor progression by reducing the production of tumor-promoting proinflammatory mediators, such as MCP-1.

## Introduction

Tumor tissues consist of a variety of cell types, including tumor cells, fibroblasts, endothelial cells, myocytes, and inflammatory cells, such as myeloid-derived suppressor cells, regulatory T cells, macrophages, and dendritic cells. The interaction of tumor cells with stromal cells leads to the production of an array of mediators that provide the soil for tumor cells to grow, invade, and metastasize. These mediators include matrix metalloproteinases, growth factors, cytokines, and chemokines (1–3).

MCP-1/CCL2 is a chemokine with potent monocyte chemotactic activity. It was initially purified from the culture supernatant of a human malignant glioma cell line (4) and a human monocytic leukemic cell line (5), and found to be identical to the previously described tumor cell-derived chemotactic factor (6). Although earlier animal studies using MCP-1-transfected tumor cells provided a combination of anti- or pro-tumor effects of MCP-1 (7–10), accumulating evidence strongly supports the notion that the production of MCP-1 by tumors promotes tumor progression. A study of a chemically induced skin papilloma showed a lower number of papillomas developing in MCP-1^−/−^ mice compared to wild type (WT) mice (11). A critical role of MCP-1 in the initiation and progression of colitis-associated colon carcinogenesis was demonstrated by using mice deficient in the MCP-1 receptor CCR2 or MCP-1 blocking agents (12). Furthermore, neutralization of MCP-1 resulted in reduced growth of prostate (13–15) and lung cancer (16), as well as reduced metastasis of breast cancer (17, 18) in mice.

In human non-small cell lung cancer (NSCLC), elevated MCP-1 production was previously reported (19, 20). Increased number of tumor infiltrating macrophages, likely regulated by increased MCP-1 production, corresponded to a shorter survival rate of NSCLC patients (19). In contrast, in another study, MCP-1 expression in cancer cells was associated with better survival in NSCLC patients (20). Recent studies using mouse NSCLC models indicated a role for MCP-1 in the progression of NSCLC. Lewis lung carcinoma (LLC) is a NSCLC cell line (21), which constitutively produces MCP-1 *in vitro* and the production can be highly upregulated in response to the TLR4 ligand LPS or TNFα (22, 23). Intrapleural injection of LLC cells induced malignant pleural effusion through MCP-1 production (22) and neutralization of MCP-1 reduced the growth of subcutaneously injected LLC cells (16). These animal studies strongly suggest a critical role of MCP-1 in the development of NSCLC. Thus, MCP-1 is a candidate molecular target of cancer treatment (24) and recent clinical trials using a neutralizing anti-MCP-1 antibody showed some anti-tumor efficacy (25, 26).

There are three potential mechanisms by which MCP-1 production is increased in tumors: (1) tumor cells constitutively produce a high level of MCP-1, (2) tumor cells produce a high level of MCP-1 in response to stimuli, and (3) stromal cells produce a high level of MCP-1 in response to stimuli, such as a tumor cell product(s). Tumor cells were originally thought to be the primary source of MCP-1 in established tumors (4–6); however, recent studies indicated that stromal cells were the primary cell source of MCP-1 in some mouse tumor transplantation models, including 4T1 breast cancer (23), M5076 sarcoma, and B16 melanoma (27).

In the present study, we aimed to examine the mechanisms of MCP-1 production in a mouse LLC transplantation model. We found that in established LLC tumors, tumor cells were the primary source of MCP-1. We further revealed that LLC cells activate macrophages to produce TNFα which, in turn, markedly increases MCP-1 production by LLC cells. Thus, crosstalk between tumor cells and stromal cells plays a major role in the production of proinflammatory, tumor-promoting mediators in a tumor microenvironment, which constitutes a plausible target for anti-cancer therapy.

## Materials and Methods

### Mice

Wild type C57BL/6 and Balb/c mice were from Charles River, Frederick, MD, USA. The generation of C57BL/6 or Balb/c MCP-1^−/−^ [MMRRC stock No. 037094-UNCC, 29S1(Cg)-Ccl2tm1.1Tyos/Mmnc] was previously described (23, 28). Myeloid cell-specific MCP-1^−/−^ mice were generated by crossing MCP-1^flox/flox^ mice (JAX Stock No. 023347, B6; 129-Ccl2 <tm1Tyos >/J) (28, 29) to LysMCre mice (30). MyD88^−/−^, TLR2^−/−^, TLR4^−/−^, TLR9^−/−^, and IL-1R1^−/−^ mice on a C57BL/6 background were from the Cancer and Inflammation Program Mouse Core, NCI, Frederick. Mouse resident peritoneal cells (PC) were obtained by flushing the peritoneal cavity of C57BL/6 mouse with 5 ml clod PBS. Mouse peritoneal exudates cells (PEC) were induced by intraperitoneal injection of 3% thioglycollate (TG) (Difco Laboratory, Detroit, MI, USA). PEC were harvested 3–4 days later by flushing the peritoneal cavity with 5 ml clod PBS. The experimental protocols of this study were approved by the Frederick National Laboratory for Cancer Research Animal Care and Use Committee, Frederick, MD, USA.

### Tumor transplantation model

LLC, 4T1, and B16F1 cells were obtained from American Type Culture Collection (ATCC) and maintained in National Cancer Institute DCTD Tumor Repository. All cell lines were tested for their mouse origin by using the Molecular Testing of Biological Materials assays by Animal Health Diagnostic Laboratory at National Cancer Institute-Frederick in 2009. LLC and 4T1 cells were cultured in RPMI 1640 (Lonza, Walkersville, MD, USA) supplemented by 10% fetal bovine serum (FBS, HyClone, Rogan, UT, USA), 100 μM glutamine, 1× penicillin/streptomycin, and 1 mM sodium pyruvate. B16F1 cells were cultured in DMEM (Lonza) supplemented by 10% FBS, 100 μM glutamine, 1× penicillin/streptomycin, and 1 mM sodium pyruvate, 1× non-essential amino acid, 1× MEM vitamins. Cells were grown to 50–80% confluence. Before injection, cells were detached with 0.2% trypsin-EDTA, washed once with medium, three times with PBS, and resuspended in PBS at 4 × 10^6^/ml for LLC and 1 × 10^6^/ml for 4T1 or B16F1 cells. One hundred microliters of cell suspension were injected into the flank for LLC or B16F1 and the mammary pad for 4T1 cells. Tumor size was measured and tumor volume was calculated using the following formula: Volume = (width)^2^ × length/2. To generate LLC tumors in the lung, 10^5^ LLC cells in 100 μl PBS were intravenously injected and tumors were harvested 2 weeks after injection.

To evaluate the level of MCP-1 mRNA expression, mice were euthanized and then tumors were excised and stored in RNAlater (Ambion). Blood was drawn by heart or mandibular puncture. Sera were isolated and stored at −80°C until use.

To recover tumor cells from tumors, tumors were excised, minced, and digested with collagenase VI (Sigma-Aldrich, St. Louis, MO, USA) for 3 h at room temperature. After removal of tissue debris, cells were rinsed with RPMI 1640 containing 10% FBS, and then plated in a tissue culture plate. Cells were passed for five generations at 1:5 before used. At this stage, the mutated MCP-1 allele was no longer detectable by PCR in tumor cells harvested from the tumor of MCP-1^−/−^ mice, indicating that there was no significant contamination by host cells.

### *In vitro* culture

One hundred thousand or 1 × 10^3^ LLC in 1 ml medium were seeded into six-well or 12-well tissue culture plates, respectively. After overnight incubation at 37°C, medium containing non-adherent cells was removed and replaced by 2 ml fresh medium. PC or PEC in 50 μl medium was added to the wells or culture insert and the plates were incubated for 2 or 5 days.

Lungs harvested from MCP-1^−/−^ mice were minced using the gentle MACS Dissociator (Miltenyi Biotec, Inc., Auburn, CA, USA), and then digested with collagenase VI for 2–3 h at room temperature. Tissue debris was removed, red blood cells were lysed, and the resulting lung cells were seeded into 12-well tissue culture plates, and then co-cultured with 1 × 10^3^ LLC cells for 5 days.

To activate tumor cells, 1 × 10^5^ tumor cells were seeded into 12-well tissue culture plates. After overnight incubation at 37°C, medium was removed and 1 ml of fresh medium was added and cells were incubated at 37°C in the presence or absence of 1 or 10 ng/ml recombinant mouse TNFα (R&D Systems, Minneapolis, MN, USA) or 100 ng/ml lipopolysaccharide (LPS, Sigma-Aldrich, St. Louis, MO, USA). Total RNA was extracted by TRIzol (Life Technologies, Grand Island, NY, USA). Cell-free culture supernatants were also prepared and kept at −80°C until use.

### Flow cytometry

After blocking Fc receptors, cells were incubated with appropriately diluted anti-mouse antibodies, including PE-F4/80 (BM8), PerCP-CD45 (30-F11), and APC-CD11b (M1/70) (Biolegend, San Diego, CA, USA). Appropriate species matched Abs served as isotype control. Acquisition of data was performed using a LSRII (BD Biosciences, Mountain View, CA, USA), and data analysis was conducted using the FlowJo software (Tree Star Inc., Ashland, OR, USA). Only live cells determined using LIVE/DEAD Fixable Dead Cell Stain Kit (Life Technologies, Grand Island, NY, USA) were analyzed.

### Northern blotting

Northern blot analysis was performed as described with a minor modification (28, 31). Filters were hybridized at 42°C overnight in ULTRAhyb (Life Technologies) with 10^6^ dpm/ml of cDNA probe labeled by ^32^P (Perkin Elmer, Cambridge, MA, USA). Filters were washed twice with 2× SSC, 0.1% SDS at room temperature for 15 min, and once with 0.1× SSC, 0.1% SDS at 60°C for 30 min prior to autoradiographic exposure.

### Gene expression profiling

The profile of gene expression was analyzed using the nCounter Analysis System (NanoString Technologies, Seattle, WA, USA) (32). The nCounter Code Set for the study contained 41 test and two control genes. The assay used two sequence-specific probes for each gene of interest. The probes were complementary to a 100-base region of the target mRNA. One probe was covalently linked to an oligonucleotide containing biotin (the capture probe); the other was linked to a color-coded molecular tag (reporter probe). Each hybridization consisted of 100 ng of total RNA, reporter, and capture probe mix for the 43 genes. The hybridization, washing, and scanning procedures were conducted according to the guidelines provided by NanoString Technologies.

### ELISA

The concentrations of MCP-1 were measured with an ELISA kit specific for mouse MCP-1 (R&D Systems). The sensitivity of the assay was 2 pg/ml.

### Statistical analysis

All experiments were performed at least twice. Results presented were from representative experiments. Statistical analysis was performed by Student’s *t*-test or log-rank (Mantel-Cox) test, using the GraphPad Prism, Version 4, 5, or 6, GraphPad Software, San Diego, CA, USA. A value of *p* < 0.05 was considered to be statistically significant.

## Results

### Activated tumor cells are the main source of MCP-1 in LLC tumors

**Figure 1:**
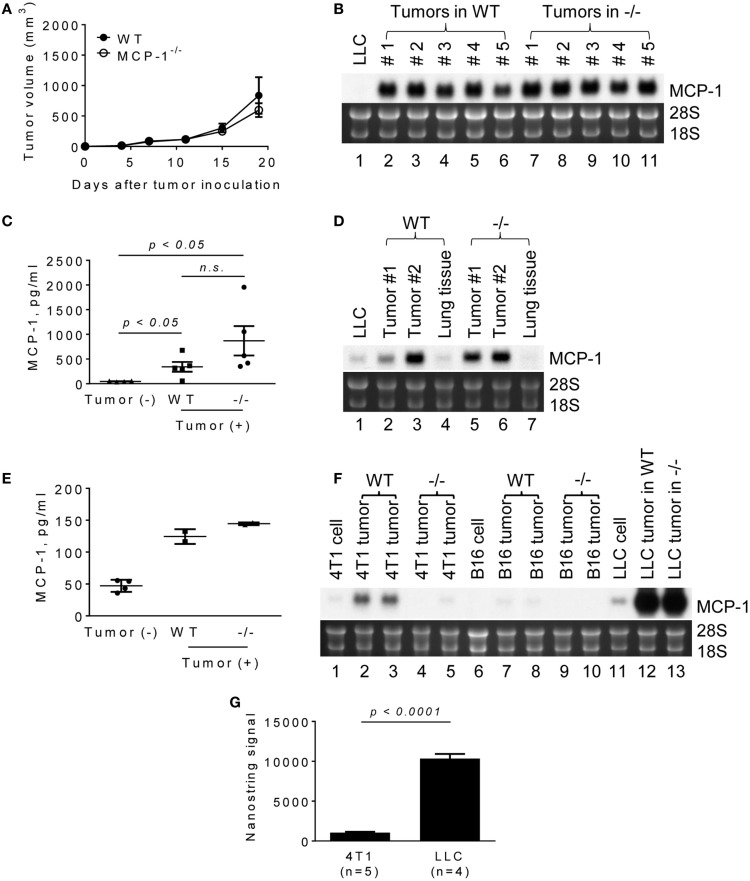
**The expression of MCP-1 in 4T1, B16, and LLC tumors growing in WT or MCP-1^−/−^ mice**. **(A)** Four hundred thousand LLC cells in 100 μl PBS were injected into the flank of female WT or MCP-1^−/−^ mice and the size of each tumor was measured and the volume was calculated. The results are shown as the mean ± SEM; *n* = 6. **(B)** The expression of MCP-1 mRNA was examined by Northern blotting 2 weeks after LLC tumor cell inoculation. **(C)** Serum MCP-1 concentrations were measured by ELISA 2 weeks after LLC tumor cell inoculation. The results are shown as the mean ± SEM. *n* = 4 for non-tumor-bearing WT mice and *n* = 5 for tumor-bearing WT or MCP-1^−/−^ mice. A summary of two experiments. **(D,E)** One hundred thousand LLC cells in 100 μl PBS were intravenously injected into WT or MCP-1^−/−^ mice. Two weeks after injection, blood was collected by heart punctured and serum was obtained. Lungs were excised and stored in RNAlater. Total RNA was extracted by TRIzol from lung tumors or adjacent lung tissues and the expression of MCP-1 mRNA expression was examined by Northern blotting. Serum MCP-1 concentration was measured by ELISA. The results are shown as the mean ± SD. *n* = 2 for tumor-bearing WT or MCP-1^−/−^ mice. **(F)** One hundred thousand 4T1 or B16 cells in 100 μl PBS were injected into the mammary pad or the flank of WT or MCP-1^−/−^ mice, respectively. Two weeks later, the expression of MCP-1 mRNA by tumors was evaluated by Northern blotting. **(G)** The expression of MCP-1 mRNA in 4T1 tumors and LLC tumors growing in WT mice was evaluated by Nanostring gene profiling. The results are shown as the mean ± SEM.

It was previously demonstrated that the blockade of MCP-1 significantly slowed the growth of primary tumors in mouse NSCLC models, including a LLC model (16). To evaluate the role for MCP-1 produced by non-tumor stromal cells in tumor growth in this model, we subcutaneously injected LLC cells into the flank of WT or MCP-1^−/−^ mice. As shown in Figure 1A, tumors grew at a similar rate in both WT and MCP-1^−/−^ mice, indicating that the lack of MCP-1 in stromal cells does not interfere with tumor. Compared to *in vitro* cultured LLC cells (Figure 1B, lane 1), much higher levels of MCP-1 mRNA were detected in all tumors from both WT (lanes 2–6) and MCP-1^−/−^ mice (lanes 7–11). Serum MCP-1 levels were also elevated in tumor-bearing WT and MCP-1^−/−^ mice (Figure 1C).

Lewis lung carcinoma is a lung cancer cell line (21). To examine whether LLC tumors formed in the lung also express high levels of MCP-1, we intravenously injected LLC cells and examined the level of MCP-1 mRNA in lung metastatic tumors 2 weeks after injection. As shown in Figure 1D, lung tumors formed in both WT and MCP-1^−/−^ mice expressed high levels of MCP-1 mRNA (lanes 2, 3, 5, 6). Since tumor tissues contained a significant amount of lung tissue that expressed only a low level of MCP-1 (lanes 4, 7), the levels of MCP-1 mRNA detected in lung tumors were lower than those in subcutaneous tumors. Serum MCP-1 levels were also elevated in both WT and MCP-1^−/−^ mice which received i.v. injection of LLC cells (Figure 1E). These results indicate that tumor cells are the primary source of MCP-1 in LLC tumors.

We then compared the levels of MCP-1 expressed in LLC tumors with those in 4T1 or B16 tumors in which stromal cells were the main source of MCP-1 (23, 27). 4T1 and B16 cells expressed lower levels of MCP-1 than LLC cells *in vitro* (Figure 1F, lanes 1, 6, 11). As we previously reported, 4T1 tumors grown in WT mice expressed moderate levels of MCP-1 mRNA (lanes 2, 3), whereas those in MCP-1^−/−^ mice expressed minimal levels of MCP-1 mRNA (lanes 4, 5). A similar observation was made for B16 tumors with much lower MCP-1 levels in tumors in WT mice (lanes 7–10). The levels of MCP-1 mRNA expressed in LLC tumors were markedly higher compared to 4T1 or B16 tumors (lanes 12, 13), with approximately 10-fold higher MCP-1 mRNA in LLC tumors than in 4T1 tumors (Figure 1G).

**Figure 2:**
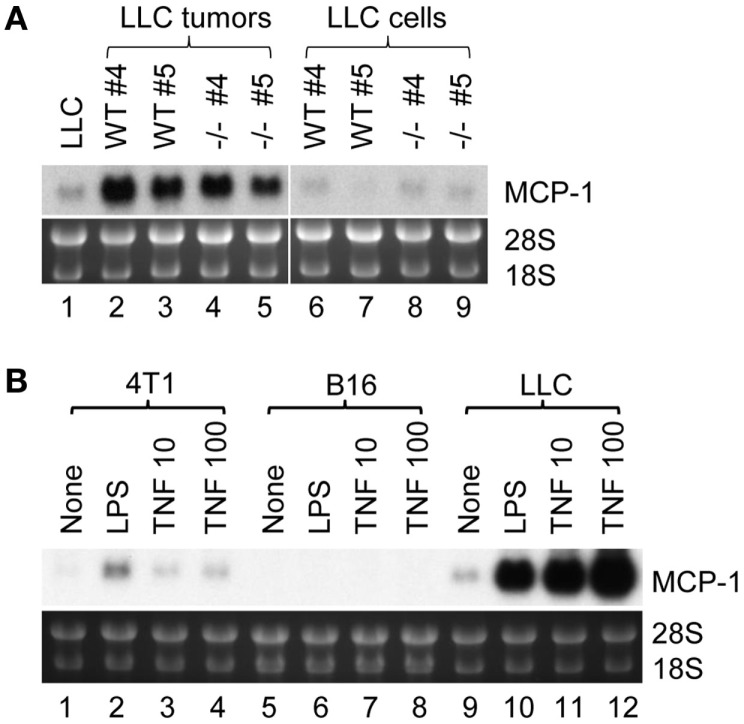
**The expression of MCP-1 in LLC cells isolated from tumors and the role of proinflammatory mediators**. **(A)** Four hundred thousand LLC cells in 100 μl PBS were injected into the flank of female WT or MCP-1^−/−^ mice. Two weeks later, mice were euthanized, and tumors were excised and cut into halves. One half was stored in RNAlater for total RNA isolation. The other half was minced and digested with collagenase VI for 3 h at room temperature. After removal of tissue debris, cells were rinsed with RPMI 1640 containing 10% FBS, and then plated in a tissue culture plate. Cells were passed for five generations at 1:5 before use. At this stage, the mutated MCP-1 allele was no longer detectable by PCR in tumor cells harvested from MCP-1^−/−^ mice, indicating that there was no significant contamination by host cells. The expression of MCP-1 mRNA was examined by Northern blotting. All samples (10 μg per lane) were loaded onto a single gel and RNA was transferred to a single membrane. **(B)** 4T1, B16, or LLC cells were stimulated by 100 ng/ml of LPS or 10 or 100 ng/ml of mouse recombinant TNFα for 6 h. Total RNA was isolated and the expression of MCP-1 mRNA was examined by Northern blotting.

There are two possibilities that could explain increased MCP-1 expression in LLC cells in tumors; one is that tumor cells constitutively express high levels of MCP-1, and the other is that tumor cells express high levels of MCP-1 in response to stimuli present in a tumor microenvironment. We, therefore, isolated LLC cells from tumors grown in either WT or MCP-1^−/−^ mice by culturing *in vitro* to deplete non-tumor cells, and examined the level of MCP-1 mRNA. As shown in Figure 2A, high levels of MCP-1 mRNA were detected in the original tumors grown in WT or MCP-1^−/−^ mice (lanes 2–5), but the levels of MCP-1 mRNA expressed by LLC cells isolated from tumors either from WT or MCP-1^−/−^ mice (mouse #4 and 5 presented in Figure 1B) were low (lanes 6–9) and comparable to that in the original LLC cells used for injection (lane 1), supporting the hypothesis that MCP-1 expression is not constitutively elevated in tumor cells, but rather, activation of tumor cells in a tumor microenvironment is the cause of elevated MCP-1 expression by tumor cells *in vivo*. In fact, LLC cells, in response to LPS or TNFα, expressed high levels of MCP-1 mRNA *in vitro* (Figure 2B, lanes 9–12). We previously reported that LLC cells produced a high level of MCP-1 protein in response to these mediators (23). Thus, there was a strong correlation between the level of MCP-1 mRNA expression and protein production. In contrast to LLC cells, 4T1 or B16 cells responded poorly to the same mediators (Figure 2B, lanes 1–8). These results indicate that LLC cells express higher levels of MCP-1, once they form tumors *in vivo* and are exposed to factors in a tumor microenvironment.

### Co-culture with macrophages markedly increases MCP-1 expression by LLC cells

**Figure 3:**
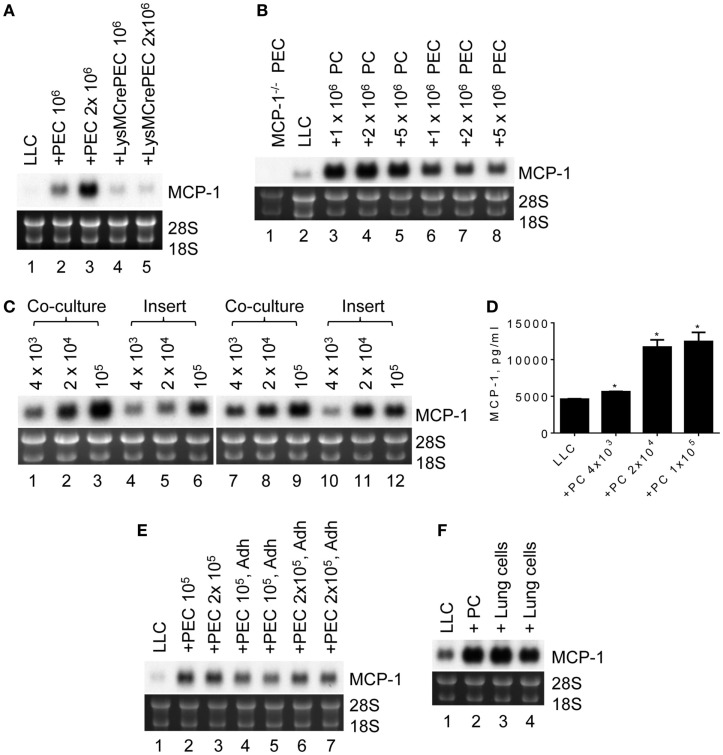
**The expression of MCP-1 in LLC cells after co-culture with peritoneal macrophages**. **(A)** One hundred thousand LLC cells were seeded into six-well plates. After overnight incubation at 37°C, 1 × 10^6^ or 2 × 10^6^ TG-induced PEC from WT or LysMCre^+^MCP-1^flox/flox^ mice were added and incubated for an additional 2 days. Total RNA was isolated and the expression of MCP-1 mRNA was examined by Northern blotting (10 μg per lane). **(B)** Ten thousand LLC were seeded into six-well plates. After overnight incubation at 37°C, 1 × 10^6^, 2 × 10^6^ or 5 × 10^6^ PC, or PEC from MCP-1^−/−^ mice were added and incubated for 5 days. Total RNA was isolated and the expression of MCP-1 mRNA was examined by Northern blotting (10 μg per lane). **(C)** One thousand LLC cells were seeded into 12-well plates. After overnight incubation at 37°C, 4 × 10^3^, 2 × 10^4^ or 1 × 10^5^ PC from WT or MCP-1^−/−^ mice were added directly to wells or into culture inserts. After incubation at 37°C for 5 days, total RNA was isolated and the expression of MCP-1 mRNA was examined by Northern blotting (10 μg per lane). **(D)** One thousand LLC cells were seeded into 12-well plates. After overnight incubation at 37°C, 4 × 10^3^, 2 × 10^4^ or 1 × 10^5^ PC from WT mice were added directly to wells. After incubation at 37°C for 5 days, the concentration of MCP-1 in the culture supernatants was measured by ELISA. The results are shown as the mean ± SEM. *p < 0.001, *n* = 4. **(E)** One hundred or two hundred thousand PEC were seeded into wells of 12-well plate and incubated at 37°C for 3 h. Non-adherent cells were removed by gently washing the wells with medium, and then 1 × 10^3^ LLC cells were added. After incubating at 37°C for 5 days, total RNA was isolated and the expression of MCP-1 mRNA was examined by Northern blotting (10 μg per lane). **(F)** One thousand LLC cells were seeded into 12-well plates. After overnight incubation at 37°C, 1 × 10^5^ lung cells from MCP-1^−/−^ mice were added directly to the wells. After incubation at 37°C for 5 days, total RNA was isolated and the expression of MCP-1 mRNA was examined by Northern blotting (10 μg per lane).

To analyze the potential mechanisms by which MCP-1 expression is elevated in LLC cells in a tumor microenvironment, we co-cultured 10^5^ LLC cells for 2 days in six-well culture plates with 10^6^ or 2 × 10^6^ PEC containing mostly inflammatory macrophages, a cellular component found in tumor microenvironment. As shown in Figure 3A, MCP-1 mRNA expression was increased in the co-cultured cells dependently of the number of PEC (lanes 1–3). However, this increase was absent when PEC from myeloid cell-specific MCP-1^−/−^ mice were used in the co-culture (lanes 4, 5), indicating that under this co-culture condition increased levels of MCP-1 mRNA were originated from activated macrophages but not LLC cells. Thus, this co-culture condition did not reflect the condition found in LLC tumors *in vivo*.

We next reduced the number of LLC cells to 10^3^ so that the culture period could be extended to 5 days. We also used resting peritoneal macrophages (PC) or TG-induced PEC from MCP-1^−/−^ to detect MCP-1 mRNA only from LLC cells. As shown in Figure 3B, there was no MCP-1 mRNA expression in PEC of MCP-1^−/−^ mice (lane 1). Interestingly, co-culture with either MCP-1^−/−^ PC or PEC increased MCP-1 mRNA expression by LLC cells (lanes 3–8) with considerably higher MCP-1 expression after co-culture with PC (lanes 3–5). One million PC or PEC were sufficient to induce the highest level of MCP-1 mRNA.

To determine whether cell contact is required for increased MCP-1 expression by LLC cells, LLC cells and PC of WT or MCP-1^−/−^ mice were either co-cultured together (Figure 3C, lanes 1–3, 7–9) or separated by a membrane (lanes 4–6, 10–12). Since PC were more efficient in increasing MCP-1 mRNA expression by LLC cells, only PC were used. Increased MCP-1 mRNA expression was detected in both culture conditions with the highest MCP-1 mRNA levels obtained with 10^5^ PC, indicating that direct cell contact is not required. There was also no difference in the level of MCP-1 mRNA detected in LLC cells co-cultured with WT (lanes 1–6) or MCP-1^−/−^ (lanes 7–12) PC, excluding the interference by macrophage-derived MCP-1 mRNA in our assay system. Consistent with the mRNA levels, there was a significant increase in MCP-1 concentration in the culture supernatants detected by ELISA when LLC cells were co-cultured with as few as 4 × 10^3^ PC (Figure 3D).

To examine whether macrophages were responsible for the increased MCP-1 mRNA expression by LLC cells, non-adherent cells were removed from TG-induced PEC by adherence and adherent cells were co-cultured with LLC cells. Both total PEC (Figure 3E, lanes 2, 3) and adherent cells in PEC (lanes 4–7) increased MCP-1 mRNA expression by LLC cells, strongly suggesting that macrophages were responsible for the increased MCP-1 expression by LLC cells.

Since LLC cells are originated from lung, we examined whether lung macrophages could also enhance MCP-1 expression by LLC cells. As shown in Figure 3F, similar to PC (lane 2), co-culture of LLC cells with MCP-1^−/−^ mouse lung cells containing an approximately 64% CD45^+^ cell population mostly macrophages (data not shown), increased MCP-1 mRNA expression by LLC cells, indicating that the interaction with mouse lung macrophages also increases MCP-1 mRNA expression by LLC cells.

### Elevated MCP-1 expression by tumor cells is dependent on macrophage TNFα

**Figure 4:**
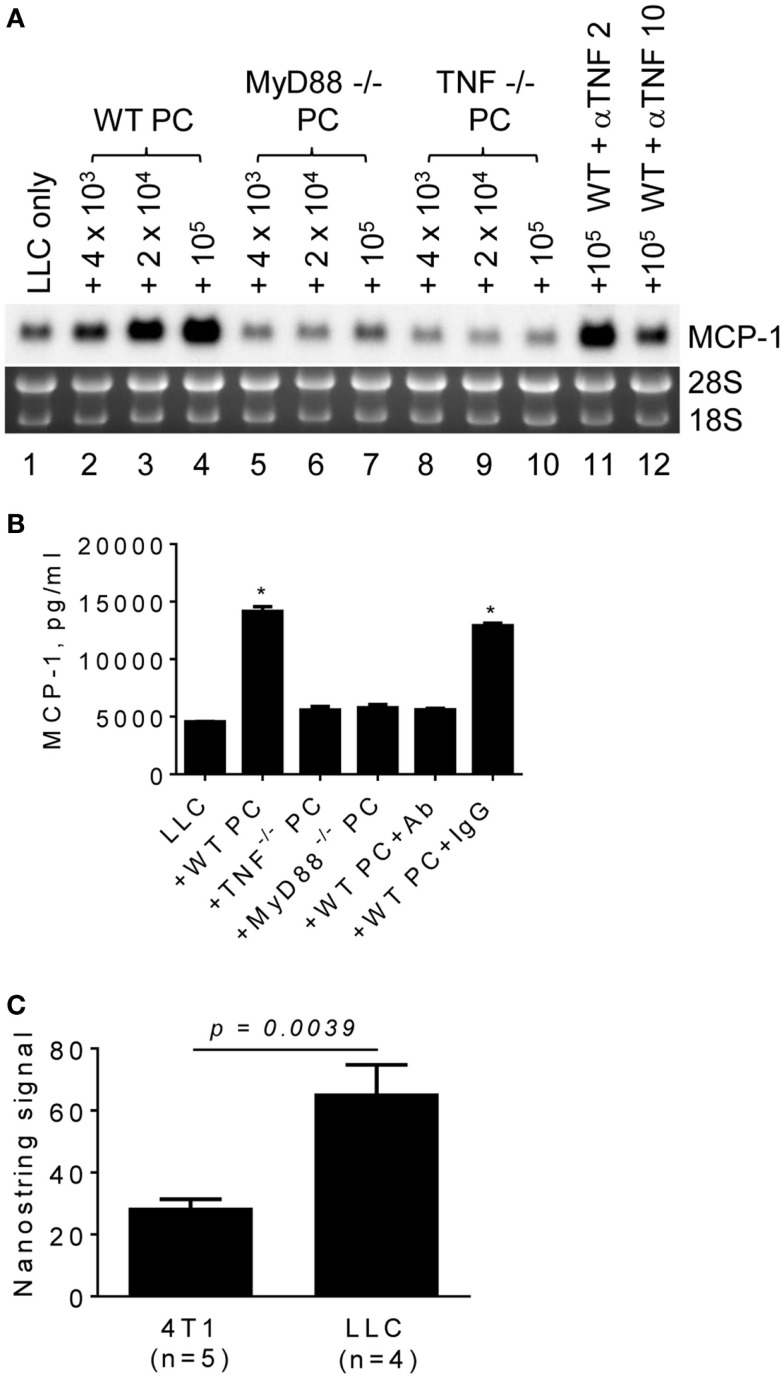
**The role of macrophage MyD88 and TNFα in MCP-1 mRNA expression by LLC cells**. **(A)** One thousand LLC cells were seeded into 12-well plates. After overnight incubation at 37°C, three different numbers of PC from WT, MyD88^−/−^ or TNF^−/−^ mice were added to the wells. To neutralize TNFα, 2 or 10 μg of anti-mouse TNFα IgG (R&D Systems) was added with 1 × 10^5^ PC from WT mouse. After incubation at 37°C for 5 days, total RNA was isolated and the expression of MCP-1 mRNA was examined by Northern blotting (10 μg per lane). **(B)** The experiment presented in *A* was repeated with 1 × 10^5^ PC from WT, MyD88^−/−^ or TNF^−/−^ mice and the MCP-1 concentration in the culture supernatants was measured by ELISA. The results are shown as the mean ± SEM. *p < 0.0001, *n* = 4. **(C)** The expression of TNF mRNA in 4T1 tumors or LLC tumors was examined by Nanostring gene profiling. The results are shown as the mean ± SEM.

TLR ligands activate macrophages and can be released from tumor cells. Those activated macrophages then produce proinflammatory cytokines, such as TNFα, in a tumor microenvironment. To examine whether these proinflammatory mediators are involved in promoting MCP-1 expression by LLC tumors, we co-cultured LLC cells with PC from MyD88^−/−^ or TNFα^−/−^ mice and examined MCP-1 mRNA expression or MCP-1 protein production by LLC cells. PC from WT mice increased MCP-1 mRNA expression by LLC cells (Figure 4A, lanes 1–4). In contrast, PC from TNFα^−/−^ mice (Figure 4A, lanes 8–10, Figure 4B) or MyD88^−/−^ mice (Figure 4A, lanes 5–7, Figure 4B) did not increase MCP-1 expression or production by LLC cells. Addition of an anti-TNFα neutralizing antibody in the co-culture of LLC cells and WT PC almost completely inhibited the effect of WT PC to promote MCP-1 mRNA expression or production by LLC (Figure 4A, lanes 11, 12, Figure 4B). The expression of TNFα mRNA was detected in both 4T1 and LLC tumors with significantly higher expression levels in LLC tumors (Figure 4C). Thus, TNFα is available in a LLC tumor microenvironment.

**Figure 5:**
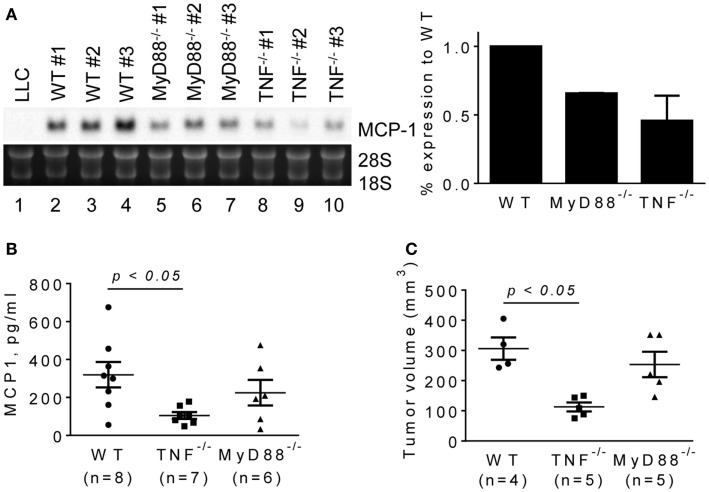
**The expression of MCP-1 mRNA in LLC tumors growing in WT, MyD88^−/−^ or TNF^−/−^ mice**. LLC cells (4 × 10^5^ cells in 100 μl PBS) were injected into the flank of female WT, MyD88^−/−^ or TNF^−/−^ mice. Two weeks later, the expression of MCP-1 mRNA in tumors, the serum MCP-1 concentrations, and the tumor volumes were analyzed. **(A)** The level of MCP-1 mRNA expression in tumors was examined by Northern blotting and quantified by densitometry. The results are shown as the mean ± SD. **(B)** Serum MCP-1 concentrations were measured by ELISA. The results are shown as the mean ± SEM. The summary of two experiments. **(C)** The volume of each tumor from each group was calculated and compared. The results are shown as the mean ± SEM.

To evaluate the role for host cell-derived TNFα and MyD88 *in vivo*, we transplanted LLC cells into the flank of WT, MyD88^−/−^ or TNFα^−/−^ mice, and examined the levels of MCP-1 mRNA in tumors after 2 weeks. Consistent with *in vitro* results, the expression of MCP-1 was markedly lower in LLC tumors grown in TNFα^−/−^ mice (Figure 5A, lanes 8–10). Serum MCP-1 levels were also lower in tumor-bearing TNFα^−/−^ mice. Importantly, the volumes of tumors in TNFα^−/−^ mice were significantly smaller than those in WT mice (Figures 5B,C). These results indicate that TNFα is a critical macrophage-derived mediator that increases the production of MCP-1 by LLC cells in tumors. Although the expression of MCP-1 was also reduced in LLC tumors grown in MyD88^−/−^ mice (Figure 5A, lanes 5–7), there was no significant difference in either serum MCP-1 level or tumor volume between MyD88^−/−^ and WT mice (Figures 5B,C).

**Figure 6:**
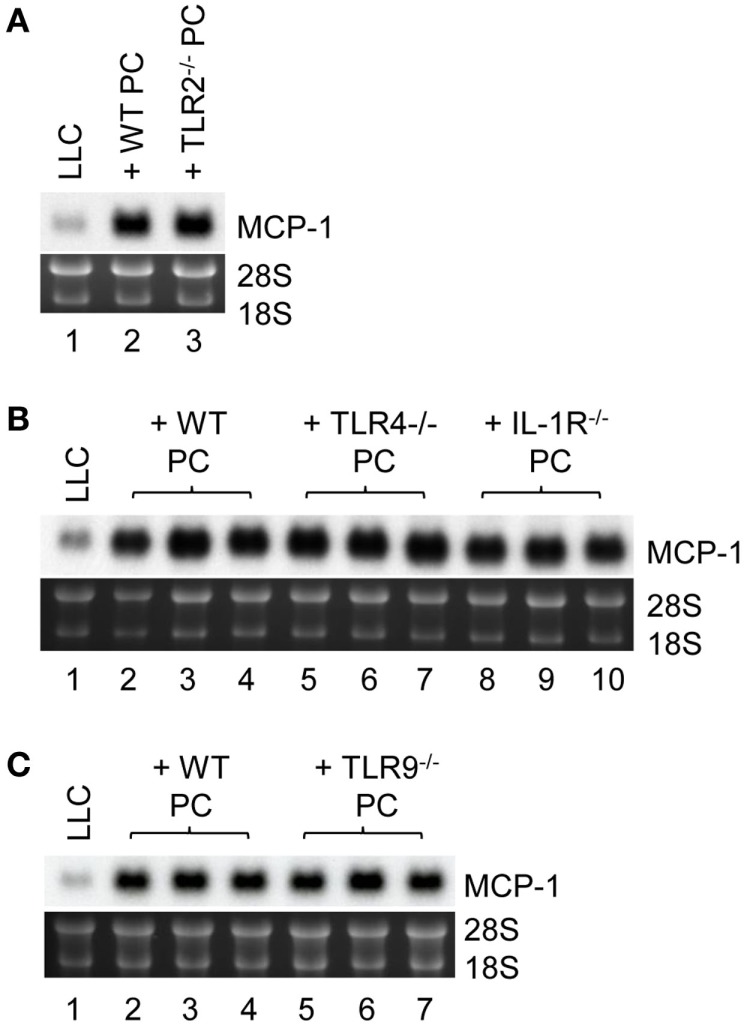
**The role of macrophage TLR2, TLR4, TLR9, and IL-1R1 in MCP-1 mRNA expression by LLC cells**. **(A)** One thousand LLC cells were seeded into 12-well plates. After overnight incubation at 37°C, 1 × 10^5^ PC from WT or TLR2^−/−^ mice were added to the wells. After incubation at 37°C for 5 days, total RNA was isolated and the expression of MCP-1 mRNA was examined by Northern blotting (10 μg per lane). **(B)** One thousand LLC cells were seeded into 12-well plates. After overnight incubation at 37°C, 1 × 10^5^ PC from WT, TLR4^−/−^ or IL-1R1^−/−^ mice were added to the wells. After incubation at 37°C for 5 days, total RNA was isolated and the expression of MCP-1 mRNA was examined by Northern blotting (10 μg per lane). **(C)** One thousand LLC cells were seeded into 12-well plates. After overnight incubation at 37°C, 1 × 10^5^ PC from WT or TLR9^−/−^ mice were added to the wells. After incubation at 37°C for 5 days, total RNA was isolated and the expression of MCP-1 mRNA was examined by Northern blotting (10 μg per lane).

Finally, we investigated the mechanistic basis for LLC cells to induce TNFα production by macrophages. It was previously demonstrated that LLC cells were able to release an extracellular matrix protein versican that activated myeloid cells to produce TNFα via TLR2 (33). MyD88 is a signaling molecule downstream of TLR2 and the loss of MyD88^−/−^ in PC reduced MCP-1 mRNA expression by LLC cells *in vitro*; therefore, TLR2 on macrophages may play a role in LLC cell-induced macrophage TNFα production. Contrary to our hypothesis, however, PC from TLR2^−/−^ mice (Figure 6A, lane 3) were as efficient as PC from WT mice (Lane 2) to increase MCP-1 expression by LLC cells. PC from TLR4^−/−^ or IL-1R1^−/−^ mice (Figure 6B, lanes 5–10) or TLR9^−/−^ mice (Figure 6C, lanes 5–7) also increased MCP-1 mRNA expression by LLC cells. Thus, it appears that LLC cells activate macrophages by a mechanism independent of TLR2, TLR4, TLR9, or IL1R1 involving MyD88. It is also possible that LLC cells activate macrophages via more than one TLR.

## Discussion

The chemokine MCP-1/CCL2 is involved in multiple stages of tumor progression, such as recruitment of immunosuppressive, tumor-promoting M2 macrophages, angiogenesis, tumor invasion, and metastasis. We and others previously demonstrated that stromal cells, but not tumor cells, were the primary source of MCP-1 in several mouse tumor models, including 4T1 breast cancer (23), MO5076 sarcoma, and B16 melanoma (27). However, since many types of tumor cells have been shown to express MCP-1 *in vitro* and *in vivo* (34), tumor cells may also be the source of MCP-1 in selected tumors. In the present study, we found that tumor cells were the primary source of MCP-1 in LLC tumors growing *in vivo*. We also revealed that LLC cells activate macrophages to produce TNFα, which in turn, further increases MCP-1 production by LLC cells. This is the first demonstration that LLC-macrophage interaction may be critical for rendering tumor cells as the primary source of MCP-1 in a tumor microenvironment.

A number of proinflammatory mediators are present in tumor microenvironments, among which TNFα is shown to promote tumor progression potentially by inducing MCP-1 production inside tumors (11, 35, 36). However, the cellular source of TNFα and the mechanisms by which TNFα is produced in tumors remain unclear. Kim et al. found that among cell lines screened, LLC cells were the most potent macrophage activators leading to the production of IL-6 and TNFα through activation of TLR2 and TLR6. Both TNFα and TLR2 in the host were required for LLC metastasis after intravenous injection of LLC cells. Biochemical analysis of LLC-conditioned medium led to the identification of an extracellular matrix proteoglycan, versican, as a macrophage activator that acts on TLR2 and its co-receptors, TLR6 and CD14 (33). Cordero et al. also demonstrated by using a fly model that TNF/Egr expressed by tumor-associated hemocytes (leukocytes in fly) was necessary and sufficient to trigger TNF signaling in tumor cells for dMMP1 expression (37). In the present study, we demonstrated that PC from TNFα^−/−^ mice did not increase MCP-1 expression by LLC cells *in vitro*. Transplantation of LLC cells into the flank of TNF^−/−^ mice resulted in reduced MCP-1 serum levels, indicating that TNF plays a critical role also *in vivo* in inducing MCP-1 production by tumor cells. Importantly, the growth of LLC tumors was slower in TNFα^−/−^ mice. A recent study by others showed that anti-MCP-1 neutralizing antibody inhibited the growth of LLC tumors (16). These findings suggest that MCP-1 is an important downstream effector molecule of TNFα involved in cancer progression.

In contrast to the role of TNFα, the role of MyD88 and TLRs in LLC-mediated macrophage activation remains unclear. Co-culture of LLC cells with PC from TLR2^−/−^, TLR4^−/−^, TNR9^−/−^ or IL-1R1^−/−^ mice increased MCP-1 mRNA expression by LLC as efficiently as WT PC. MyD88-deficiency in macrophages reduced MCP-1 expression and production by LLC cells *in vitro*, but MyD88-deficiency did not reduce serum MCP-1 levels or LLC tumor volumes as effectively as TNFα-deficiency *in vivo*. These results suggest that other TLRs may mediate LLC-induced macrophage TNFα production, and MyD88-independent mechanisms may also play a role *in vivo*. Additional studies are necessary to more precisely define the role of TLR signaling in the activation of macrophages by LLC cells.

LLC cells were highly responsive to the TLR4 ligand LPS or TNFα to express high levels of MCP-1. A human NSCLC cell line, A549, also expresses and produces a high level of MCP-1 in response to TNFα (38) (confirmed by our own study). In contrast, 4T1 breast cancer and B16 melanoma cells responded poorly to the same stimuli and expressed only a low level of MCP-1. The expression of TNFα was found in both 4T1 and LLC tumors; thus, TNFα is available in both tumors and capable of stimulating tumor cells to produce high levels of MCP-1. These findings support the hypothesis that the crosstalk between tumor and stromal cells is tightly controlled by factors present in tumor microenvironments and these factors and the responsiveness of tumor cells may determine the primary source of MCP-1 in each tumor.

It should be noted that in the present study, we used a tumor transplantation model in which LLC cells were subcutaneously injected into the flank of mice. In addition, we used i.v. injection of LLC cells as a model of lung tumors. Therefore, our results, while they are valid, need to be interpreted with caution and further validated in other tumor models, in particular in orthotopic tumor implantation models, to dissect the crosstalk between tumor and stromal cells in producing tumor-promoting chemokines, such as MCP-1. Recently, Weiss et al. reported an orthotopic model of LLC (also known as 3LL) tumor in which very small numbers of tumor cells were directly injected into the lung parenchyma to generate a solitary pulmonary nodules (39). Resulting lung tumors were surrounded by normal lung parenchyma that grew locally with the infiltration of fibroblasts and myeloid cells, such as CD45^+^Gr-1^med^CD11b^high^ myeloid-derived suppressor cells, over time. Interestingly, the expression and production of MCP-1 and MCP-3/CCL7 were markedly upregulated in the lung tumors in this model. In our study, we also detected the upregulation of MCP-1 expression in lung tumors formed by intravenously injected LLC cells despite a relatively small number of tumor specimen examined. Thus, it is important to further analyze the mechanisms by which lung cancer cells interact with stromal cells for tumor progression using a clinically relevant orthotopic model and additional cancer cell lines.

Naturally arising tumors are heterogeneous and may vary in their capacity to respond to stimuli and produce mediators to support tumor progression. Our study provided a novel mechanism whereby tumors produce a high level of tumor-promoting, proinflammatory mediators, such as MCP-1, in response to TNFα released by macrophages, in tumor microenvironments. Better understanding the complex interaction of tumor cells with cells in stroma, such as macrophages, may provide opportunities to develop additional anti-cancer therapies.

## Conflict of Interest Statement

The authors declare that the research was conducted in the absence of any commercial or financial relationships that could be construed as a potential conflict of interest.
